# HLA-DRB1 and HLA-DQB1 Are Associated with Adult-Onset Immunodeficiency with Acquired Anti-Interferon-Gamma Autoantibodies

**DOI:** 10.1371/journal.pone.0128481

**Published:** 2015-05-26

**Authors:** Manop Pithukpakorn, Ekkapong Roothumnong, Nasikarn Angkasekwinai, Bhoom Suktitipat, Anunchai Assawamakin, Voravich Luangwedchakarn, Pinklow Umrod, Wanna Thongnoppakhun, Suporn Foongladda, Yupin Suputtamongkol

**Affiliations:** 1 Division of Medical Genetics, Department of Medicine, Faculty of Medicine Siriraj Hospital, Mahidol University, Bangkok, Thailand; 2 Division of Molecular Genetics, Department of Research and Development, Faculty of Medicine Siriraj Hospital, Mahidol University, Bangkok, Thailand; 3 Division of Infectious Diseases and Tropical Medicine, Department of Medicine, Faculty of Medicine Siriraj Hospital, Mahidol University, Bangkok, Thailand; 4 Department of Biochemistry, Faculty of Medicine Siriraj Hospital, Mahidol University, Bangkok, Thailand; 5 Department of Pharmacology, Faculty of Pharmacy, Mahidol University, Bangkok, Thailand; 6 Department of Immunology, Faculty of Medicine Siriraj Hospital, Mahidol University, Bangkok, Thailand; 7 Department of Microbiology, Faculty of Medicine Siriraj Hospital, Mahidol University, Bangkok, Thailand; Cambridge University, UNITED KINGDOM

## Abstract

Recently a newly identified clinical syndrome of disseminated non-tuberculous mycobacterial diseases (with or without other opportunistic infections in adult patients who were previously healthy, has been recognized in association with an acquired autoantibody to interferon-gamma. This syndrome is emerging as an important cause of morbidity and mortality, especially among people of Asian descent. Trigger for the production of this autoantibody remains unknown, but genetic factors are strongly suspected to be involved. We compared HLA genotyping between 32 patients with this clinical syndrome, and 38 controls. We found that this clinical syndrome was associated with very limited allele polymorphism, with HLA-DRB1 and DQB1 alleles, especially HLA-DRB1*15:01, DRB1*16:02, DQB1*05:01 and DQB1*05:02. Odds ratio of DRB1*15:01, DRB1*16:02, DQB1*05:01 and DQB1*05:02 were 7.03 (95% CI, 2.18–22.69, P<0.0001, 9.06 (95% CI, 2.79–29.46, P<0.0001), 6.68 (95% CI, 2.29–19.52, P = 0.0004), and 6.64 (95% CI, 2.30–19.20, P = 0.0004), respectively. Further investigation is warranted to provide better understanding on pathogenesis of this association.

## Introduction

Nontuberculous mycobacterial (NTM) diseases have been increasingly diagnosed worldwide [1]. Recently a newly identified clinical syndrome of disseminated NTM disease (with or without other opportunistic infections such as salmonellosis, penicillosis, and varicella-zoster virus infection) in adult patients who previously healthy, has been recognized in association with an acquired autoantibody to interferon-gamma (IFN- γ) [2]. The clinical features of these patients are similar to those with genetic defects in the IFN- γ/IL-12 pathway in that they present with progressive or disseminated infection with mycobacteria of low virulence. Their clinical responses to antimycobacterial therapy are unsatisfactory with either slow remission or relapses, despite administration of appropriate drugs and good patient compliance [3].

This clinical syndrome is emerging as an important cause of morbidity and mortality, especially among Asian descent [2]. Although trigger for the production of this autoantibody remains unknown, genetic factors are strongly suspected to be involved. Numerous studies have demonstrated that HLA genes, are linked or play an important role in pathogenesis of many autoimmune diseases, adverse drug reactions and increased susceptibility to certain infections [4–8]. A recent clinical study of 18 patients from Taiwan indicated that HLA-DRB1*16:02 and DQB1*05:02 are associated with this syndrome [9]. This report prompted us to investigate a cohort of 32 patients with this clinical syndrome, diagnosed and followed up at Faculty of Medicine Siriraj Hospital, Mahidol University, Bangkok, Thailand.

## Methods

### Study population

All patients were adults (18 years old and above) and followed up for their infections at Faculty of Medicine Siriraj Hospital between 2003 and 2013. Case was defined as previously healthy and non-HIV infected patients diagnosed with either disseminated NTM disease or other opportunistic infections (such as disseminated penicillosis, salmonellosis) or both, whom autoantibody to IFN- γ was detected. Control composed mainly of anonymous healthy blood donors. We also included 6 non-HIV infected patients who presented with culture-proven active pulmonary tuberculosis as control in order to compare the HLA genotype between two distinct mycobacterial infections. All controls did not have detectable autoantibody to IFN- γ in their sera. Disseminated disease was defined as infection in at least two noncontiguous, sterile sites. All cases and controls had no history of cancer, immunodeficiency, or immunosuppression within 4 weeks before enrollment or diagnosis of their infections. All participants provided written informed consent according to the study protocol, which was approved by the Siriraj Institutional Review Board. The study was conducted according to the Good Clinical Practice and the Declaration of Helsinki.

At baseline or enrollment, complete histories were obtained and physical examinations with routine clinical laboratory tests were performed in all participants. Demographic data, place of birth, detailed characteristics of their infections such as site of infection, causative organism, clinical courses and outcome of treatment were also recorded. Data of cases were recorded on standardized case-report forms.

Reactive skin diseases associated with this syndrome were diagnosed as described previously into sweet syndrome, acute generalized exanthematous pustulosis (AGEP), pustular psoriasis and erythema nodosum [3]. Sweet syndrome featured a high-grade fever with generalized erythematous, variable-sized, papules or plaques. Blood test showed polymorphonuclear leukocytosis, typically over 10,000/mm^3^ [10]. The outcome of treatment in this study was clinically categorized into three groups; remission, stable and active disease. Remission was defined as the absence of any new symptom and sign after discontinuation of antibiotic treatment for at least 6 months. Stable disease was defined as resolution of fever, but incomplete resolution of other symptoms and signs, with concurrent use of oral antibiotic therapy, and active disease was defined as worsening of symptoms and signs which required intravenous antibiotic retreatment within the last 3 months of followed up. Six milli-litre of EDTA blood were collected from all participants for DNA extraction. Other clinical specimens were obtained if clinically indicated.

### Anti-IFN- γ autoantibody assay

Antibodies to IFN- γ in serum were measured by enzyme-linked immunosorbant assay (ELISA) modified from methods previously described by Ding L, et al [11]. Average Optical density (OD) resulted from duplicate wells was reported. OD of at least 1 is considered positive in this hospital. This cut-off OD was selected according to the in-house data comparing between a group of patients with this syndrome and more than 100 healthy controls.

### DNA Preparation and PCR Amplification

Genomic DNA was isolated using the Gentra Puregene blood kit (Qiagen USA) according to the manufacturer’s instructions. DNA concentration and quality were determined using a Nanodrop ND-1000 spectrophotometer (Thermo Scientific, USA) and diluted with TE low EDTA buffer (10mM Tris-HCl, 0.1mM EDTA) to a concentration of 50 ng/μL.

Each genomic DNA sample was amplified within 96 well PCR plate of GS G-Type HLA MR&HR Primer Set (Roche/454 Life Sciences, Branford, USA) designed for use with 454 Sequencing systems, enabling high-resolution typing and unambiguous allele assignment of class I and class II loci of the HLA genes in a single run (a total of 14 amplicons of HLA-A, B, C, DQA1, DQB1 and DRB1-3-4-5).

### Amplicon Preparation (Purification, Quantification, Normalization, and Pooling)

The amplicons were then quantified by Quant-iT PicoGreen dsDNA Reagent and Kits (Invitrogen, UK) on an Infinite 200 PRO microplate reader (TECAN, Switzerland) using the i-control v1.9 software. Serial dilution of eight standard DNA concentrations used to create a standard curve (with linear regression, r^2^ was ranging from 0–100 ng/well). Thereafter amplicons were normalized by diluting to an appropriate concentration of 1x10^9^ pooled PCR amplicons and were purified using Agencourt AMPure XP paramagnetic beads (Beckman Coulter, USA) using a 1:1 ratio of DNA: bead. The purified libraries were further quantified by the Quant-iT PicoGreen dsDNA Assay Kit (Invitrogen), normalized and equimolar pooled to be 1x10^8^ pool of each sample was 10-fold serially diluted to get the final concentration of 1x10^5^ being pooled to the final eight samples per pool (a total of 400 μl from which 50 μl was used as a template for further emulsion PCR).

### Emulsion PCR and Sequencing (EmPCR, Emulsion Breaking, Bead Enrichment: Wash & Capture, Pyrosequencing)

Emulsion PCR, oil breaking, and bead enrichment were performed using the GS Junior Titanium emPCR Kit (Lib-A), emPCR Reagents Lib-A kit, Oil and Breaking Kit, and the Bead Recovery Reagents Kit according to the manufacturer’s instructions (Roche/454 Life Sciences, Branford, USA). Sequencing was carried out using the GS Junior Titanium Sequencing Kit and the GS Junior system (Roche/454 Life science, Branford, USA) following the manufacturer’s instructions without modifications.

### HLA Data analysis

High-resolution HLA genotypes were called with Assign ATF 454 software v1.1.0.33 (Conexio Genomics, Pty Ltd, Perth, Australia) which compares the DNA sequence reads obtained in FASTA format (.fna) to the Assign ATF 454 HLA references. HLA genotyping was done with next-generation sequencing platform using 454 GS Junior System (454 Life Sciences). HLA allele analysis and call was done with Assign ATF 454 (Conexio Genomics) software.

### Statistical analysis of HLA association

HLA allele frequency was calculated as a count of number of alleles divided by the total number of alleles presented in all samples. Frequencies of HLA alleles in Thai population were calculated among the control group. Associations between HLA allele and case group were performed using Chi-square test comparing the presence of a specific HLA allele among cases versus control. A nominal significant level with multiple-testing correction, corresponded to a p-value of 0.05/[total number of alleles], was used to determine a statistically significant association. The effects of each HLA alleles on anti-IFN- γ antibody were estimated using an odds ratio, adjusting for age, sex, and additional covariates related to opportunity infection susceptibility. HLA alleles with frequency less than 10 percent were grouped together as a rare HLA allele, and were tested for association with NTM infection as a group.

## Results

### Clinical presentations of cases

A total of 32 cases were enrolled of which 18 (56.2%) were female and the median age was 50.5 years (range, 24–74 years). The cases were from various regions of Thailand (9 patients were from the northern region, 7 patients were from the northeastern region, 9 patients were from the central region, four patients were from the southern region, two patients were from the western region and one patient was from the eastern region). Their demographic characteristics, proven pathogens and affected sites (listed in order of occurrence) and the outcome of treatment are summarized in Table 1. All patients had no prior medical condition, with one exception who had hypothyroidism after subtotal thyroidectomy. Overall the patients had 76 episodes of opportunistic infections. Eighteen patients (56.2%) had NTM disease as their first presentation. Two patients had salmonella infection as their sole causative pathogen. *Mycobacterium abscessus* was the most common causative pathogen (25 of 76) and NTM (25 of 39 NTM) identified, followed by salmonellosis (15 of 76) and varicella zoster virus infection (10 of 76). Twenty-three patients (71.8%) had more than 1 episode of opportunistic infection. All patients with *M*. *abscessus* infection presented with fever, multiple cervical or generalized lymphadenopathy and weight loss. Twenty patients (62.5%) had 27 episodes of reactive skin diseases, most of which were sweet syndrome (15 cases), followed by AGEP (4 cases). Most patients (81.2%) had active disease (6 cases) or persistent infection (20 cases) requiring long term oral antimicrobial therapy. Four patients (12.5%) died from their uncontrolled infections and only 2 patients (6.2%) went into remission without taking any antimicrobial agents.

**Table 1:** Demographic characteristics, pathogen, affected site and outcome of patients with disseminated non-tuberculous mycobacterial infections or other opportunistic infections who had positive anti-IFN- γ autoantibody.

Patients	Age(yr)/ Gender	Birthplace	Pathogen (affected site)	Outcome
1	43/F*	Central	*M. tuberculosis* *** (tissue biopsy), *Salmonella* spp. (blood), *M*. *abscessus* (blood)	Stable
2	57/F	North	*Histoplasma capsulatum* (LN****), *Penicillium marneffei* (synovial fluid), *Burkholderia pseudomallei* (LN), *M*. *abscessus* (LN), *Salmonella* gr. B (pus at leg)	Death
3	53/M**	Central	*M*. *abscessus* (LN), *M*. *intracellulare* (blood and sputum), Varicella zoster virus infection (clinical)	Stable
4	37/F	NE	*M*. *abscessus* (LN), *Salmonella* spp. (blood)	Death
5	57/F	NE	*M*. *abscessus* (LN)	Stable
6	48/F	Central	*Salmonella* gr. C (blood)	Stable
7	48/F	North	*M*. *abscessus* (bone marrow)	Stable
8	69/M	North	*Salmonella* gr. D (bone marrow), *M*. *hemophilum* (blood)	Stable
9	57/F	NE	*M*. *abscessus* (LN, sputum)	Stable
10	60/F	North	*M*. *fortuitum* (LN), Varicella zoster virus infection (clinical), *Penicillium marneffei* (skin), *M*. *abscessus* (blood)	Stable
11	26/M	North	*M*. *abscessus* (LN), *Cryptococcus neoformans* (CSF), *M*. *fortuitum* (LN), *Penicillium marneffei* (joint, tissue at shoulder)	Death
12	51/M	North	*Salmonella* spp. (blood), *M*. *abscessus* (LN), Varicella zoster virus infection (clinical)	Remission
13	68/M	South	*M*. *abscessus* (LN), Varicella zoster virus infection (clinical)	Stable
14	57/M	NE	*M*. *abscessus* (LN, blood, skin), *Salmonella* gr. D (blood), *Hemophilus influenzae* (sputum, blood)	Death
15	24/M	South	*M*. *abscessus* (LN, blood, transbronchial biopsy)	Stable
16	74/M	South	*M*. *scrofulaceum* (tissue at face), *M*. *fortuitum* (LN)	Stable
17	46/F	Central	*M*. *avium* complex (LN), Varicella zoster virus infection (clinical)	Stable
18	59/M	North	*M*. *abscessus* (LN)	Remission
19	33/F	Central	*Salmonella* gr. C (blood), Varicella zoster virus infection (clinical), *M*. *abscessus* (LN)	Active disease
20	47/M	Central	*Burkholderia pseudomallei* (blood), *Salmonella* gr. C (Psoas abscess), *Mycobacterium* spp. (LN)	Stable
21	41/F	NE	*Salmonella* gr. C (blood, subdural and Psoas abscess)	Active disease
22	51/M	NE	Varicella zoster virus infection (clinical), *Salmonella* gr. C (blood), *M*. *avium* complex (blood)	Active disease
23	36/F	East	*Cryptococcus neoformans* (blood, BAL***** fluid), *M*. *abscessus* (blood, LN)	Stable
24	42/F	NE	*Salmonella* gr. B (brain abscess), *M*. *fortuitum* (blood), *M*. *abscessus* (blood)	Stable
25	40/M	South	Varicella zoster virus infection (clinical), *Salmonella* spp. (blood), *Burkholderia pseudomallei* (splenic abscess), *M*. *abscessus* (blood)	Stable
26	64/M	West	*Mycobacterium* spp. (LN)	Active disease
27	58/F	North	*M*. *intracellulare* (pus at shoulder), *M*. *abscessus* (LN)	Active disease
28	64/M	Central	*Salmonella* gr. D (blood), *M*. *abscessus* (LN)	Stable
29	43/F	Central	*M*. *fortuitum* (blood, LN), *Mycobacterium* spp. (clinical)	Stable
30	50/F	Central	*M*. *scrofulaceum* (blood, bone marrow, tissue), *Mycobacterium* spp. (clinical)	Stable
31	63/F	West	*M*. *abscessus* (LN)	Stable
32	46/F	North	*Salmonella* gr. B (blood), *Cryptococcus neoformans* (blood, joint fluid), *M*. *abscessus* (blood)	Stable

*F = Female

**M = Male

***M. = *Mycobacterium*

****LN = Lymph node

*****BAL = Bronchoalveolar lavage.

There were 36 participants enrolled as controls, of which 30 were normal healthy controls while another 6 were patients with culture-proven active pulmonary tuberculosis. A total of 62.5% of the controls were female and the median age of the cohort was 45 years (range, 21–62 years).

### HLA genotyping

From the data of HLA class I, we found that HLA-A*02, A*11 and A*24. HLA-B*13, B*15, B*46, B*56, C*01, C*03, C*04 and C*07 were the prominent HLA-A, HLA-B and HLA-C alleles respectively in both cases and controls. There was no statistically significant difference observed amongst HLA class I alleles between case and control groups.

Interestingly, for HLA class II, we found that HLA-DRB1*15 and DRB1*16 accounted for 82.8% of total HLA-DRB1 alleles in patient group, whereas those combined alleles only equaled 20.8% in the control group (Tables 2 and 3). In fact, all patients carried at least one HLA-DRB1*15 or DRB1*16 allele or both. HLA-DRB1*15, DRB1*16 and DQB1*05 were found more frequently, while DQB1*03:01 and DQB1*03:03 were found less frequently in patient group as compared to the control group. HLA-DRB1*16:01 and DRB1*16:09 were found only in case group. We found that 27 out of 32 patients carried at least one HLA-DRB1*16 alleles (19 patients had DRB1*16:02, 4 patients had DRB1*16:01, 5 patients had DRB1*16:09 and 1 patient had both DRB1*16:01 and DRB1*16:02). Twenty-four out of 32 patients carried at least one HLA-DRB1*15 alleles (17 patients had DRB1*15:01, 7 patients had DRB1*15:02). Four patients who did not have DRB1*16 instead were having DRB1*15:01 allele. The presence of HLA-DRB1*15 or DRB1*16 alleles showed strongly significant association with disseminated opportunistic infection with anti- IFN- γ antibody (OR 10.5, 95% CI 3.42–32.33, and OR 33.48, 95% CI 8.74–128.21, respectively) (Tables 4 and 5). Among DRB1*15 and DRB1*16 that were found in case group, the odds ratio of DRB1*15:01 and DRB1*16:02 were 7.03 (95%CI 2.18–22.69) and 9.06 (95%CI 2.79–29.46) respectively. Because DRB1*16:01 and DRB1*16:09 were not found in control group, the odds ratio of both DRB1 alleles could not be calculated.

**Table 2:** HLA class II genotypes of 32 affected individuals with disseminated opportunistic infection and positive anti- IFN- γ autoantibody (case group).

Case ID	HLA-DRB1	HLA-DRB1	HLA-DQB1	HLA-DQB1	Case ID	HLA-DRB1	HLA-DRB1	HLA-DQB1	HLA-DQB1
1	15:01	16:02	05:02	05:02	17	14:141	15:01	05:01	05:03
2	15:01	16:02	05:01	05:02	18	12:02	15:01	03:01	05:01
3	12:02	16:02	03:01	05:02	19	15:02	16:09	05:01	05:02
4	15:01	16:02	05:02	05:02	20	15:02	15:02	05:01	05:01
5	15:01	16:02	05:01	05:02	21	15:02	16:09	05:01	06:02
6	14:09	15:01	05:01	05:02	22	15:02	16:02	05:02	05:02
7	16:01	16:02	05:02	05:02	23	15:01	16:02	03:01	05:02
8	15:01	16:09	05:01	05:02	24	15:02	16:02	05:01	05:02
9	15:01	16:01	05:01	05:01	25	15:02	16:09	05:01	05:01
10	15:01	16:02	05:01	05:02	26	15:02	16:02	05:01	05:02
11	15:01	16:01	05:01	05:01	27	13:01	16:02	02:01	05:02
12	12:02	16:02	02:01	05:02	28	08:09	16:02	04:02	05:02
13	09:01	16:02	03:03	05:02	29	10:01	16:02	05:01	05:02
14	15:01	16:01	05:01	05:01	30	15:01	16:02	05:01	05:02
15	15:01	16:09	05:01	05:02	31	15:01	16:02	05:01	05:02
16	14:01	16:02	05:02	05:02	32	01:01	15:01	05:01	05:01

**Table 3:** HLA class II genotypes of healthy volunteers (No. 1–30) and pulmonary tuberculosis (No. 31–36) without anti- IFN- γ autoantibody (control group).

Control ID	HLA-DRB1	HLA-DRB1	HLA-DQB1	HLA-DQB1	Control ID	HLA-DRB1	HLA-DRB1	HLA-DQB1	HLA-DQB1
1	12:20	14:141	03:01	05:02	19	03:01	09:01	02:01	03:03
2	10:01	12:02	03:01	05:01	20	14:01	16:02	05:02	05:02
3	07:01	09:01	02:01	03:03	21	16:02	16:02	03:02	05:02
4	13:11	14:141	03:01	05:02	22	11:21	11:89	02:02	03:01
5	09:05	15:01	03:02	03:03	23	04:09	12:02	03:01	03:02
6	14:01	15:01	05:02	05:02	24	12:02	15:02	03:01	06:01
7	03:38	12:02	03:01	03:01	25	09:01	09:01	02:02	03:03
8	14:46	16:02	05:02	05:02	26	14:01	14:01	04:02	05:03
9	08:09	09:01	03:03	04:02	27	15:01	15:01	05:01	05:02
10	04:05	08:03	04:01	04:05	28	10:01	10:04	04:01	05:01
11	09:01	09:05	03:03	03:03	29	08:03	08:09	04:02	04:04
12	12:02	11:01	03:01	03:01	30	12:02	16:02	03:01	05:02
13	10:01	10:01	03:02	05:01	31	12:02	15:01	03:01	05:01
14	09:01	09:02	03:03	03:03	32	08:09	12:01	03:01	04:02
15	12:02	14:141	05:02	05:02	33	04:01	04:01	04:03	04:05
16	08:03	15:01	05:01	05:01	34	09:01	16:02	03:03	05:02
17	08:04	14:141	03:01	03:01	35	14:01	15:02	05:01	05:03
18	14:04	14:04	04:01	05:03	36	08:03	15:02	05:01	05:01

**Table 4:** Number of individuals who carried HLA-DRB1 and DQB1 subtypes between patients and control, and odds ratio of each subtype.

HLA	Number		Odds ratio	95% CI	P value
	Case (N = 32)	Control (N = 36)			
DRB1*15	**24**	**8**	**10.5**	**3.42–32.23**	**1.37x10** ^**-6**^
DRB1*16	**27**	**5**	**33.48**	**8.74–128.21**	**3.08x10** ^**-9**^
DRB1*16:02	**19**	**5**	**9.06**	**2.79–29.46**	**9.66x10** ^**-5**^
DRB1*15:01	**17**	**5**	**7.03**	**2.18–22.69**	**6.08x10** ^**-4**^
DRB1*15:02	7	3	3.08	0.72–13.12	0.109
DRB1*12:02	3	8	0.36	0.09–1.51	0.134
DRB1*08:09	1	3	0.35	0.04–3.60	0.353
DRB1*10:01	1	3	0.35	0.04–3.60	0.353
DRB1*14:01	1	4	0.26	0.03–2.44	0.217
DRB1*14:14	1	4	0.26	0.03–2.44	0.217
DQB1*05:01	**21**	**8**	**6.68**	**2.29–19.52**	**3.27x10** ^**-4**^
DQB1*05:02	**23**	**10**	**6.64**	**2.30–19.20**	**2.98x10** ^**-4**^
DQB1*05:03	1	3	0.35	0.04–3.60	0.352
DQB1*03:01	3	10	0.27	0.07–1.08	0.051
DQB1*03:03	1	7	0.008	0.001–0.07	1.05x10^-11^
DQB1*02:01	2	1	2.33	0.20–27.03	0.455
DQB1*04:02	1	4	0.26	0.03–2.44	0.217

**Table 5:** Number of individuals who carried both HLA-DRB1*15:01or 16:02 and DQB1*05:01 or 05:02 between patients and control, and odds ratio of each haplotype.

HLA DRB1 HLA DQB1	Number		Odds ratio	95% CI	P value
	Case (N = 32)	Control (N = 36)			
DRB1*16:02 DQB1*05:01	8	0	N/A	N/A	N/A
DRB1*16:02 DQB1*05:02	19	4	11.69	3.33–41.07	2.76x10^-5^
DRB1*15:01 DQB1*05:01	14	2	13.22	2.70–64.71	2.16x10^-4^
DRB1*15:01 DQB1*05:02	10	2	7.73	1.54–38.66	6.25x10^-3^

For HLA-DQB1, DQB1*05 accounted for 87.5% of total HLA-DQB1 alleles in patient group, whereas control group had those combined alleles equal to 33.3%. All patients had at least one HLA-DR*05 allele, 23 out of 32 patients had DQB1*05:02 and 21 out of 32 patients had DQB1*05:01 allele. DQB1*05:03 was found in one patient. Among DQB1*05 that was found in case group, the odds ratio of both DQB1*05:01 and DQB1*05:02 alleles were 6.68 (95%CI 2.29–19.52) and 6.64 (2.3–19.2) respectively (Table 4). The multiple loci analysis of HLA-DRB1 and DQB1 also demonstrated similarly significant association with the disease (Table 5). There was no significant change in the results when the pulmonary tuberculosis group was excluded from analysis. The HLA allele frequencies of control group (S1–S4 Tables) were comparable to other Thai cohorts [12, 13].

## Discussion

Recent studies showed a high prevalence of autoantibody to IFN- γ in previously healthy non-HIV infected adult Thai patients with disseminated NTM disease [2]. Subgroup of 10 patients who were followed up at our hospital, and all anonymous healthy blood donors from that study, were included in this genetic study. In addition we identified and included 6 patients with active pulmonary tuberculosis as controls. All cases in this study had detectable autoantibody to IFN- γ. The first presentation of patients was either NTM disease, especially those caused by *M*. *abscessus*, non-typhoidal salmonellosis or varicella zoster virus infection, although one patient in this study presented with extra-pulmonary tuberculosis. During follow up, most of them developed multiple episodes of opportunistic infections, with either the same or with a different causative organism. We observed that treatment of only their opportunistic infections was associated with unsatisfactory outcomes, such as recurrent, persistent of their initial infections, or subsequent development of other opportunistic infections. Four of the patients died despite appropriate antimicrobial treatment. This observation suggested that more long-term follow up studies are needed to determine whether their autoantibodies or their levels change with treatment or resolution of infection. Studies to determine risk factors associate with their clinical course and outcome, and potential intervention are also urgently needed.

The role of HLA in several immune disorders has been described. Many autoimmune diseases such as rheumatoid arthritis, ankylosing spondylitis and type 1 diabetes are associated with specific HLA genotypes [7, 14]. Stevens-Johnson syndrome and toxic epidermal necrolysis related to carbamazepine and allopurinol are also associated with specific HLA genotypes amongst Asian population [4, 5]. In this study, we confirmed that this clinical syndrome was associated with limited allele polymorphism. HLA-DRB1*15, DRB1*16 and DQB1*05 subgroups, especially DRB1*15:01, DRB1*15:02, DRB1*16:02, DQB1*05:01 and DQB1*05:02 were the common alleles observed among cases. We found that all cases carried either one of those HLA-DRB1 or DQB1 alleles or both. It is known that HLA-DRB1 and HLA-DQB1 has strong linkage disequilibrium. So it would be difficult to make a firm conclusion on which genetic locus has more significant association with the disease. The comprehensive HLA genotyping throughout class I and II was performed to ensure that no additional HLA group is strongly associated with this condition.

**Fig 1:**
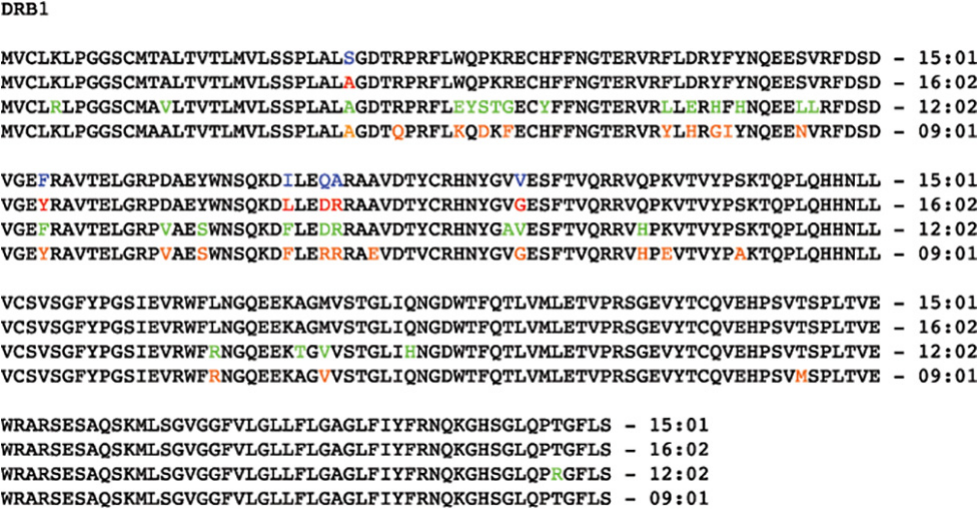
Amino acid sequence alignment of various HLA-DRB1 subtypes.

**Fig 2:**
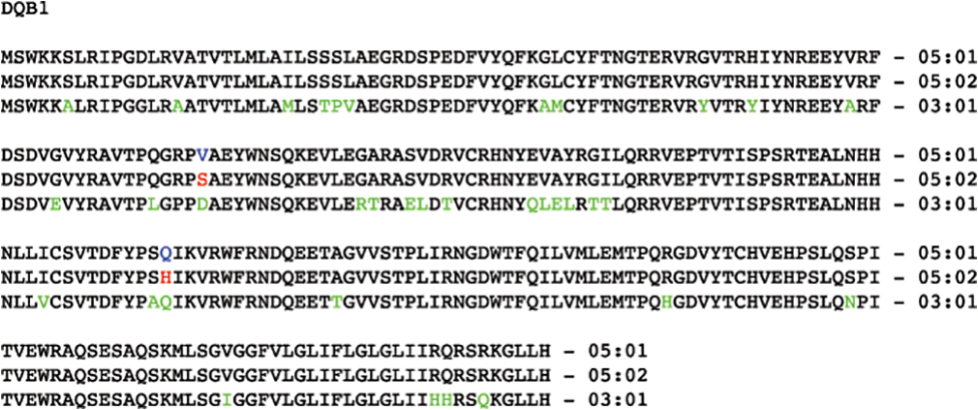
Amino acid sequence alignment of various HLA-DQB1 subtypes.

In addition to the strong association with DRB1*16:02 and DQB1*05:02 similarly found in the Taiwanese cohort, HLA-DRB1*15:01 and DQB1*05:01 were also associated with this syndrome in our Thai cohort. HLA-DRB1*16:01 and DRB1*16:09 were found only in case group. This data may suggest that HLA-DRB1*15 and DRB1*16 subgroup, rather than each of those specific alleles, is associated with this condition. It is noted that HLA-DRB1*16:02 and DQB1*05:02 are more prevalent in Asian than Caucasian population. However, HLA-DRB1*15:01 and DQB1*05:01 are found to be as common among Caucasian and African American population as Thai population (http://www.allelefrequencies.net) [15]. Various reasons could explain the difference in HLA-DRB1 and DQB1 genotypes between Taiwan and Thai cohorts. First, susceptibility to many autoimmune diseases are known to be associated with multiple HLA alleles. For example, HLA-DRB1*04:01, 04:04, 04:05, 01:01 and 10:10 alleles contribute to rheumatoid arthritis susceptibility [16]. HLA-DQB1*03:02, 03:03 and 02:01 alleles are associated with type 1 diabetes [17]. Second, the HLA-class II, especially HLA-DRB1, is highly diverse and has among the highest sequence variation in human genome. From 266 amino acids incorporate into HLA-DRB1 peptides, HLA-DRB1*15 and DRB1*16 variants are mostly identical with few amino acid discrepancies. For example, there are only 6 different amino acids between HLA-DRB1*15:01 and DRB1*16:02 (Fig 1). This observation is also noticed in HLA-DQB1 which DQB1*05:01 and DQB1*05:02 has only 2 different amino acids out of 261 amino acid peptide (Fig 2). HLA-DR and HLA-DQ together are expressed mainly on the surface of antigen presenting cells and recognized by T cell receptors. It is possible that those HLA-DRB1*15 and DRB1*16 variants or HLA-DQB1*05:01 and DQB1*05:02 variants may have comparable effect in antigen presentation process and pathogenesis of anti-IFN- γ antibody production. Finally, there could be the third locus in close proximity to HLA-DRB1 and DQB1 loci that could be another contributing cause of the disease. Although our study indicated the strong link between multiple HLA-DRB1 and DQB1 and acquired anti-IFN- γ antibody, the genetic association alone are not sufficient to trigger the disease. Many potential mechanisms underlie associated HLA alleles with the autoimmunity. HLA may play a causative role by involving disease-triggering antigenic presentation. HLA could also have an influence on Treg cells, resulting in autoreactivity. Therefore, it is likely that an unidentified factor is required to create the autoimmunity in those individuals who carry susceptible HLA alleles.

In conclusion, this study is the largest cohort of patients that demonstrated a strong association between HLA-DRB1 and DQB1 alleles, especially HLA-DRB1*15:01, DRB1*16:02, DQB1*05:01 and DQB1*05:02, and disseminated opportunistic infection with acquired anti-IFN-γ autoantibody. The mechanism to explain this association remains unknown. Further investigation is warranted to provide better understanding on pathogenesis of this syndrome.

## Supporting Information

S1 Table Allele frequencies of HLA class I, including HLA-A, HLA-B, and HLA-C among 32 cases and 30 healthy controls. (DOCX)

S2 Table Allele frequencies of HLA-DRB1 among 32 cases and 30 healthy controls. (DOCX)

S3 Table Allele frequencies of HLA-DPA1 and HLA-DPB1 among 32 cases and 30 controls. (DOCX)

S4 Table Allele frequencies of HLA-DQA1 and HLA-DQB1 among 32 cases and 30 controls. (DOCX)

## References

[1] Brode SK, Daley CL, Marras TK. The epidemiologic relationship between tuberculosis and non-tuberculous mycobacterial disease: a systematic review. Int J Tuberc Lung Dis. 2014;18(11):1370–7. Epub 2014/10/10. 10.5588/ijtld.14.0120 . 25299873

[2] Browne SK, Burbelo PD, Chetchotisakd P, Suputtamongkol Y, Kiertiburanakul S, Shaw PA, et al Adult-onset immunodeficiency in Thailand and Taiwan. N Engl J Med. 2012;367(8):725–34. Epub 2012/08/24. 10.1056/NEJMoa1111160 . 22913682 PMC4190026

[3] Chetchotisakd P, Kiertiburanakul S, Mootsikapun P, Assanasen S, Chaiwarith R, Anunnatsiri S. Disseminated nontuberculous mycobacterial infection in patients who are not infected with HIV in Thailand. Clin Infect Dis. 2007;45(4):421–7. Epub 2007/07/20. CID50631 [pii] 10.1086/520030 . 17638188

[4] Tassaneeyakul W, Jantararoungtong T, Chen P, Lin PY, Tiamkao S, Khunarkornsiri U, et al Strong association between HLA-B*5801 and allopurinol-induced Stevens-Johnson syndrome and toxic epidermal necrolysis in a Thai population. Pharmacogenet Genomics. 2009;19(9):704–9. Epub 2009/08/22. 10.1097/FPC.0b013e328330a3b8 . 19696695

[5] Chen P, Lin JJ, Lu CS, Ong CT, Hsieh PF, Yang CC, et al Carbamazepine-induced toxic effects and HLA-B*1502 screening in Taiwan. N Engl J Med. 2011;364(12):1126–33. Epub 2011/03/25. 10.1056/NEJMoa1009717 . 21428768

[6] Lionetti E, Castellaneta S, Francavilla R, Pulvirenti A, Tonutti E, Amarri S, et al Introduction of gluten, HLA status, and the risk of celiac disease in children. N Engl J Med. 2014;371(14):1295–303. Epub 2014/10/02. 10.1056/NEJMoa1400697 . 25271602

[7] Thomas GP, Brown MA. Genetics and genomics of ankylosing spondylitis. Immunol Rev. 2010;233(1):162–80. Epub 2010/03/03. 10.1111/j.0105-2896.2009.00852.x IMR852 [pii]. . 20192999

[8] Blackwell JM, Jamieson SE, Burgner D. HLA and infectious diseases. Clin Microbiol Rev. 2009;22(2):370–85. Epub 2009/04/16. 10.1128/CMR.00048-0822/2/370 [pii]. 19366919 PMC2668228

[9] Chi CY, Chu CC, Liu JP, Lin CH, Ho MW, Lo WJ, et al Anti-IFN-gamma autoantibodies in adults with disseminated nontuberculous mycobacterial infections are associated with HLA-DRB1*16:02 and HLA-DQB1*05:02 and the reactivation of latent varicella-zoster virus infection. Blood. 2013;121(8):1357–66. Epub 2012/12/18. 10.1182/blood-2012-08-452482 blood-2012-08-452482 [pii]. . 23243276

[10] Wallach D, Vignon-Pennamen MD. From acute febrile neutrophilic dermatosis to neutrophilic disease: forty years of clinical research. J Am Acad Dermatol. 2006;55(6):1066–71. Epub 2006/11/14. S0190-9622(06)02082-2 [pii] 10.1016/j.jaad.2006.07.016 . 17097401

[11] Ding L, Mo A, Jutivorakool K, Pancholi M, Holland SM, Browne SK. Determination of human anticytokine autoantibody profiles using a particle-based approach. J Clin Immunol. 2012;32(2):238–45. Epub 2011/12/16. 10.1007/s10875-011-9621-8 . 22170314

[12] Romphruk AV, Romphruk A, Kongmaroeng C, Klumkrathok K, Paupairoj C, Leelayuwat C. HLA class I and II alleles and haplotypes in ethnic Northeast Thais. Tissue Antigens. 2010;75(6):701–11. Epub 2010/03/17. 10.1111/j.1399-0039.2010.01448.x TAN1448 [pii]. . 20230525

[13] Romphruk AV, Puapairoj C, Romphruk A, Barasrux S, Urwijitaroon Y, Leelayuwat C. Distributions of HLA-DRB1/DQB1 alleles and haplotypes in the north-eastern Thai population: indicative of a distinct Thai population with Chinese admixtures in the central Thais. Eur J Immunogenet. 1999;26(2–3):129–33. Epub 1999/05/20. . 10331158 10.1046/j.1365-2370.1999.00133.x

[14] Tsai S, Santamaria P. MHC Class II Polymorphisms, Autoreactive T-Cells, and Autoimmunity. Front Immunol. 2013;4:321 Epub 2013/10/18. 10.3389/fimmu.2013.00321 24133494 PMC3794362

[15] Gonzalez-Galarza FF, Christmas S, Middleton D, Jones AR. Allele frequency net: a database and online repository for immune gene frequencies in worldwide populations. Nucleic Acids Res. 2011;39(Database issue):D913–9. Epub 2010/11/11. 10.1093/nar/gkq1128 gkq1128 [pii]. 21062830 PMC3013710

[16] Pratesi F, Petit Teixeira E, Sidney J, Michou L, Puxeddu I, Sette A, et al HLA shared epitope and ACPA: just a marker or an active player? Autoimmun Rev. 2013;12(12):1182–7. Epub 2013/08/21. 10.1016/j.autrev.2013.08.002 S1568-9972(13)00147-X [pii]. . 23958703

[17] Stankov K, Benc D, Draskovic D. Genetic and epigenetic factors in etiology of diabetes mellitus type 1. Pediatrics. 2013;132(6):1112–22. Epub 2013/11/06. 10.1542/peds.2013-1652 peds.2013-1652 [pii]. . 24190679

