# Supplementary material for: HLA-DRB1 and HLA-DQB1 Are Associated with Adult-Onset Immunodeficiency with Acquired Anti-Interferon-Gamma Autoantibodies
Source: PLoS One. 2015 May 26;10(5):e0128481. doi: 10.1371/journal.pone.0128481 (PMC4444022; doi:10.1371/journal.pone.0128481)
Supplement: S1 Table — (DOCX) [file pone.0128481.s001.docx]

**S1 Table. Allele frequencies of HLA class I, including HLA-A, HLA-B, and HLA-C among 32 cases and 30 healthy controls.**

| HLA-A | Case | Control | HLA-B | Case | Control | HLA-C | Case | Control |
| --- | --- | --- | --- | --- | --- | --- | --- | --- |
| 01:01 | 0 | 0.017 | 07:02 | 0 | 0.05 | 01:02 | 0.156 | 0.183 |
| 02:01 | 0.016 | 0.1 | 07:05 | 0.016 | 0.017 | 03* | 0.219 | 0.167 |
| 02:03 | 0.172 | 0.083 | 08:01 | 0.031 | 0 | 04* | 0.156 | 0.067 |
| 02:06 | 0.047 | 0 | 13:01 | 0.125 | 0.05 | 06:02 | 0 | 0.050 |
| 02:07 | 0.063 | 0.083 | 15* | 0.125 | 0.133 | 07:01 | 0.000 | 0.033 |
| 02:101 | 0 | 0.017 | 18* | 0.047 | 0.017 | 07:02 | 0.281 | 0.250 |
| 02:130 | 0.016 | 0.017 | 27* | 0.078 | 0.05 | 07:04 | 0.047 | 0.033 |
| 02:148 | 0.047 | 0.017 | 35* | 0.047 | 0.083 | 07:24 | 0.031 | 0.033 |
| 02:152 | 0 | 0.017 | 37* | 0 | 0.033 | 07:28 | 0 | 0.017 |
| 02:171 | 0.016 | 0 | 38:02 | 0.141 | 0.05 | 08* | 0.063 | 0.083 |
| 02:213 | 0.016 | 0 | 40* | 0.125 | 0.217 | 12* | 0.016 | 0.067 |
| 03* | 0.016 | 0.033 | 46:01 | 0.125 | 0.15 | 14* | 0.016 | 0.033 |
| 11* | 0.297 | 0.283 | 48:01 | 0 | 0.017 | 15* | 0.031 | 0.017 |
| 24* | 0.188 | 0.167 | 50:01 | 0 | 0.017 |  |  |  |
| 26:09 | 0 | 0.017 | 51:01 | 0.031 | 0.05 |  |  |  |
| 31* | 0.016 | 0.017 | 52:01 | 0.031 | 0.017 |  |  |  |
| 33:03 | 0.016 | 0.1 | 54:01 | 0 | 0.017 |  |  |  |
| 34* | 0.047 | 0.017 | 56* | 0.047 | 0 |  |  |  |
| 68* | 0.016 | 0.033 | 58:01 | 0.016 | 0.017 |  |  |  |
| 74:01 | 0.016 | 0 |  |  |  |  |  |  |
