# Supplementary material for: HLA-DRB1 and HLA-DQB1 Are Associated with Adult-Onset Immunodeficiency with Acquired Anti-Interferon-Gamma Autoantibodies
Source: PLoS One. 2015 May 26;10(5):e0128481. doi: 10.1371/journal.pone.0128481 (PMC4444022; doi:10.1371/journal.pone.0128481)
Supplement: S2 Table — (DOCX) [file pone.0128481.s002.docx]

**S2 Table. Allele frequencies of HLA-DRB1 among 32 cases and 30 healthy controls.**

| HLA-DRB1 | Case | Control | HLA-DRB1 | Case | Control |
| --- | --- | --- | --- | --- | --- |
| 01:01 | 0.016 | 0 | 11:01 | 0 | 0.017 |
| 03:01 | 0 | 0.017 | 11:21 | 0 | 0.017 |
| 03:38 | 0 | 0.017 | 11:89 | 0 | 0.017 |
| 04:05 | 0 | 0.017 | 12:02 | 0.047 | 0.117 |
| 04:09 | 0 | 0.017 | 14:01 | 0.016 | 0.067 |
| 07:01 | 0 | 0.017 | 14:04 | 0 | 0.033 |
| 08:03 | 0 | 0.05 | 14:09 | 0.016 | 0 |
| 08:04 | 0 | 0.017 | 14:14 | 0.016 | 0.067 |
| 08:09 | 0.016 | 0.033 | 14:46 | 0 | 0.017 |
| 09:01 | 0.016 | 0.117 | 15:01 | 0.266 | 0.083 |
| 09:02 | 0 | 0.017 | 15:02 | 0.125 | 0.017 |
| 09:05 | 0 | 0.033 | 16:01 | 0.063 | 0 |
| 10:01 | 0.016 | 0.067 | 16:02 | 0.297 | 0.083 |
| 10:04 | 0 | 0.017 | 16:09 | 0.078 | 0 |
