# Supplementary material for: HLA-DRB1 and HLA-DQB1 Are Associated with Adult-Onset Immunodeficiency with Acquired Anti-Interferon-Gamma Autoantibodies
Source: PLoS One. 2015 May 26;10(5):e0128481. doi: 10.1371/journal.pone.0128481 (PMC4444022; doi:10.1371/journal.pone.0128481)
Supplement: S3 Table — (DOCX) [file pone.0128481.s003.docx]

**S3 Table. Allele frequencies of HLA-DPA1 and HLA-DPB1 among 32 cases and 30 healthy controls.**

| HLA-DPA1 | Case | Control | HLA-DPB1 | Case | Control |
| --- | --- | --- | --- | --- | --- |
| 01:01 | 0.328 | 0.183 | 01:01 | 0.016 | 0.017 |
| 01:02 | 0.406 | 0.183 | 02* | 0.078 | 0.183 |
| 01:03 | 0 | 0.067 | 03* | 0.016 | 0.083 |
| 02:01 | 0 | 0.067 | 04* | 0.078 | 0.133 |
| 03:01 | 0.016 | 0.267 | 05* | 0.391 | 0.367 |
| 04:01 | 0.016 | 0.033 | 10:01 | 0.016 | 0.017 |
| 05:01 | 0.031 | 0.1 | 13:01 | 0.297 | 0.117 |
| 06:01 | 0.047 | 0.1 | 14:01 | 0.031 | 0 |
|  |  |  | 19:01 | 0.016 | 0.017 |
|  |  |  | 21:01 | 0.078 | 0.183 |
|  |  |  | 26:01 | 0.016 | 0.083 |
|  |  |  | 31:01 | 0.078 | 0.133 |
