# Supplementary material for: HLA-DRB1 and HLA-DQB1 Are Associated with Adult-Onset Immunodeficiency with Acquired Anti-Interferon-Gamma Autoantibodies
Source: PLoS One. 2015 May 26;10(5):e0128481. doi: 10.1371/journal.pone.0128481 (PMC4444022; doi:10.1371/journal.pone.0128481)
Supplement: S4 Table — (DOCX) [file pone.0128481.s004.docx]

**S4 Table. Allele frequencies of HLA-DQA1 and HLA-DQB1 among 32 cases and 30 healthy controls.**

| HLA-DQA1 | Case | Control | HLA-DQB1 | Case | Control |
| --- | --- | --- | --- | --- | --- |
| 01:01 | 0.328 | 0.183 | 02:01 | 0.031 | 0.033 |
| 01:02 | 0.406 | 0.183 | 02:02 | 0 | 0.033 |
| 01:03 | 0 | 0.067 | 03:01 | 0.047 | 0.217 |
| 02:01 | 0 | 0.067 | 03:02 | 0 | 0.067 |
| 03:01 | 0.016 | 0.267 | 03:03 | 0.016 | 0.15 |
| 04:01 | 0.016 | 0.033 | 04:01 | 0 | 0.05 |
| 05:01 | 0.031 | 0.1 | 04:02 | 0.016 | 0.05 |
| 06:01 | 0.047 | 0.1 | 04:04 | 0 | 0.017 |
|  |  |  | 05:01 | 0.422 | 0.1 |
|  |  |  | 05:02 | 0.438 | 0.217 |
|  |  |  | 05:03 | 0.016 | 0.033 |
|  |  |  | 06:01 | 0 | 0.017 |
|  |  |  | 06:02 | 0 | 0.017 |
